# Special Issue “Emerging Arboviruses”

**DOI:** 10.3390/v13030467

**Published:** 2021-03-12

**Authors:** Remi N. Charrel

**Affiliations:** Unité des Virus Emergents, Aix Marseille University, IRD 190, INSERM U1207, 13005 Marseille, France; remi.charrel@univ-amu.fr

The emergence and re-emergence of arboviruses have occurred for centuries. One of the most common vectors for arboviruses is *Aedes aegypti*, or the yellow fever mosquito. As well as yellow fever, this species can spread dengue, chikungunya, Zika and Mayaro. Due to the slave trade in Africa and rising globalisation, the range of *A. aegypti* expanded dramatically throughout the 15th to 19th centuries. This resulted in many dengue fever epidemics that spread through Asia, Africa and North America in the 18th and 19th centuries. Before 1970 just nine countries had experienced serious dengue epidemics, but the disease is now endemic in over 100 countries. For more than 30 years after WWII, the Rockefeller foundation has constituted the perfect environment for studying the mechanisms of emergence and to operate longitudinal surveillance for selected arboviruses combined with ecological inventories.

After having been for a long time the emblematic viruses threatening humanity at the turn of the 19th century and during most of the 20th century, arthropod-borne have been progressively neglected in favour of newcomers such as the hepatitis viruses (HBV and HCV) and HIV which, contrary to arboviruses, cause chronic infections. Then for decades, arboviruses were considered, at least in developed countries, as playing a second role in global health. The notion itself of “emerging viruses” was seen as outdated, particularly in high Gross Domestic Product countries.

From 1999, the West Nile virus invasion of the United States and soon after of other countries in the Americas marked the return of arboviruses as major human health pathogens in industrialized countries and showed that the impact of emerging pathogens goes beyond public health issues. Afterwards, the dispersion of arboviruses has occurred very rapidly due to the intensive growth of global transportation systems, arthropod adaptation to urbanization, failure to contain mosquito populations and land perturbation.

The same scenario repeated soon after. For 50 years, Chikungunya virus was confined to sub-Saharan Africa and Southeast Asia. The situation changed abruptly in 2005–2006 with epidemics in Indian Ocean Islands and adaptation to *Aedes albopictus* mosquito; ten years later, Chikungunya virus had invaded the Americas and caused millions of cases.

Zika virus, first identified in 1947 in Africa, remained almost unnoticed until 2007 when an outbreak hit Yap Pacific Island, after which there was another invasion of the Americas with a massive epidemic during which unravelled pathogenesis and clinical forms were described with microcephaly and neurological disorders.

The last decade has witnessed a large number of epidemics due to arboviruses causing human and animal outbreaks. There is globalization tendency of arboviruses with large epidemics touching frequently several continents.

Therefore, improving our capacities for response is a major challenge. Preparedness and response programs must be encouraged, and gaps in capacities must be identified and corrected. These programs have to provide the medical community with detection tools applicable in routine diagnostic laboratories to enable rapid detection of cases and to monitor in a timely manner the unpredictable dissemination of emerging pathogens.

The scope of this series of articles is deliberately limitless, not only with regard to the type of arbovirus considered, but also with regard to their capacity to cause diseases of medical or veterinary interest, or even if they have not yet demonstrated an established pathogenesis. Any aspects of research were also considered as eligible such as epidemiology, development and evaluation of diagnostic assays, transmission pathways and cycles, natural cycles, virulence and clinical aspects. Studies aiming at virus discovery or at establishing the pathogenesis of viruses for either humans or animals are also within the scope of this Special Issue. Special interest was also awarded to studies in the framework of Preparedness and Response activities.

